# Protic Ionic Liquids in Contrast to Aprotic Analogs: Transport Properties and Electrochemical Reactivity

**DOI:** 10.1002/tcr.202500351

**Published:** 2026-05-11

**Authors:** Masayoshi Watanabe, Seiji Tsuzuki

**Affiliations:** ^1^ Advanced Chemical Energy Research Center, Institute of Advanced Sciences Yokohama National University Kanagawa Japan

**Keywords:** fuel cell, hydrogen bond, ionic liquid, ionicity, polyimide, protic ionic liquid, proton transfer

## Abstract

Since ionic liquids (ILs) are fluids composed entirely of ions, their transport properties differ fundamentally from those of conventional electrolyte solutions containing nonionic solvents. Ionicity serves as a useful metric for characterizing IL transport behavior, defined as the ratio between the experimentally measured conductivity and that calculated from the pulsed‐field gradient (PFG) NMR diffusivities of cations and anions using the Nernst–Einstein equation. In this account, we review the ionicity of aprotic ILs and discuss how its magnitude depends on the chemical structures of constituent ions, considering directionally correlated ionic motion. We then contrast the ionicity and thermal stability of protic ILs, prepared via proton‐transfer reactions between Brønsted acids and bases, with those of aprotic counterparts. Protic ILs are characterized by strong hydrogen‐bonding interactions between cations and anions and by relatively low potential barriers for back proton transfer. Finally, the reactivity of labile protons in protic ILs is exploited in the context of their function as proton‐conducting, nonaqueous electrolytes, and their potential application as fuel cell electrolytes is discussed.

## Graphical abstract

The transport properties and electrochemical reactivity of protic ionic liquids (ILs) are compared with those of aprotic analogs. Protic ILs are characterized by strong cation–anion hydrogen bonding and labile protons, enabling proton conduction and highlighting their potential as fuel cell electrolytes.

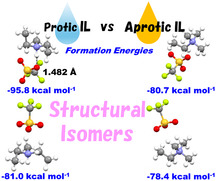

## 1 Introduction

Ionic liquids (ILs) are defined as molten salts having melting points (*T*_m_) lower than 100°C, and most of which are organic salts with a high degree of structural tunability. Since pure ILs are composed of two chemical species (i.e., a cation and an anion), the number of possible ILs corresponds to the product of the numbers of available cations and anions. For instance, 10 different cations and 10 different anions can yield 100 structurally distinct ILs. ILs are recognized as a third class of solvents and electrolytes, following water (aqueous electrolytes) and organic solvents (organic electrolytes), and are distinguished by unique properties such as negligible volatility, low flammability, high thermal stability, and high ionic conductivity [1, 2, 3]. The wide liquid temperature range (typically up to 400°C) with minimal vapor pressure and negligible weight loss has attracted considerable attention, which contrasts with a solvent such as water having a liquid range of 100°C, but still it has a wide liquid range in conventional liquids. ILs also provide an environment with extremely high ionic concentrations, and in some cases, nanoscale structural organization is observed.

Active study on ILs accelerated after Wilkes et al. reported water‐ and oxygen‐stable ILs in 1992 [4]. It is now well known that certain combinations of organic cations, such as imidazolium, quaternary ammonium, and pyridinium derivatives, and bulky and soft anions, including [PF_6_]^−^, [BF_4_]^−^, [CF_3_SO_3_]^−^, [(CF_3_SO_2_)_2_N]^−^, and [(FSO_2_)_2_N]^−^, form ILs near room temperature [1, 2, 3, 4]. These ILs are classified as “aprotic” ILs (AILs), as no active protons are incorporated into their structures. Before this development, the major room‐temperature ILs were chloroaluminate‐based systems, consisting of AlCl_4_^−^ and Al_2_Cl_7_^−^ anions paired with imidazolium and pyridinium cations [5, 6, 7]. These ILs are formed through Lewis‐acid (AlCl_3_)/Lewis‐base (Cl^−^) chemistry, and organic reactions and electrodeposition in these chloroaluminate ILs were major area of study. A serious limitation of these ILs is their instability toward water and oxygen, particularly water, which motivated the development of water‐ and oxygen‐stable ILs and subsequent intensive research. However, the history of room‐temperature ILs extends further back. In 1914, well before Wilkes et al., Paul Walden reported the synthesis of ethylammonium nitrate, the first known room‐temperature molten salt (*T*_m_ = 12°C) [8]. This work laid the foundation for modern IL research and their wide range of applications. This IL is classified as “protic” ILs (PILs) [3, 9, 10], as active protons (e.g., C_2_H_5_N*H*_3_^+^) are incorporated into their structures. PILs can also be prepared by partial neutralization of polyprotic acids, such as H_2_SO_4_ and H_3_PO_4_. For example, half‐neutralized H_2_SO_4_ salts can form protic ILs containing *H*SO_4_^−^ anions with active protons [9, 10].

**FIGURE 1:**
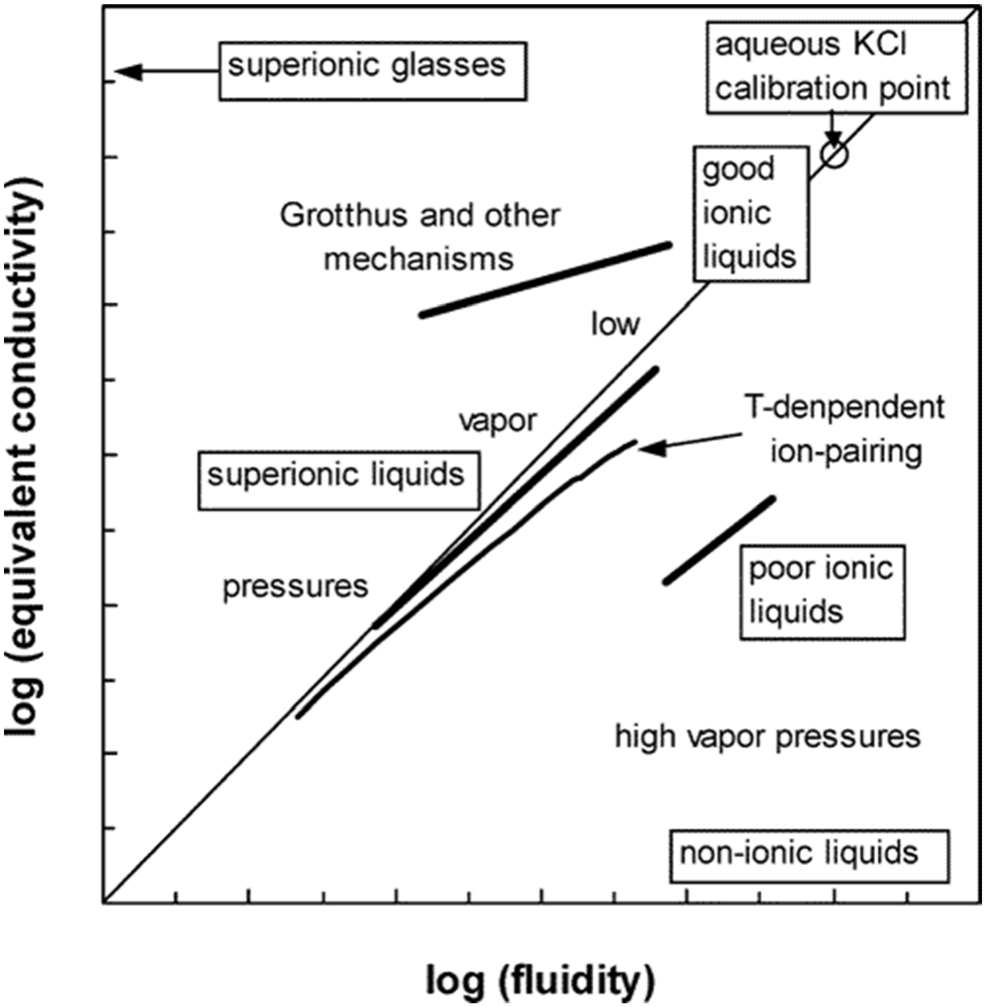
Classification diagram for ionic liquids, based on the classical Walden rule, and deviations therefrom. Reproduced from Ref. [12] Copyright (2003) with permission from the American Chemical Society.

In addition to the classification of ILs into aprotic and protic types, Angell et al. proposed a classification based on Walden plots [11, 12], which relate the logarithm of molar conductivity (log *Λ*) to the logarithm of fluidity (log *η* ^−1^, where *η* is viscosity). Using the Walden plot of an ideal aqueous strong electrolyte solution (1 M KCl) as a reference, an ideal line is drawn representing *Λη* = constant (Figure 1) [12]. ILs are then classified according to their deviation from this ideal line into “good,” “poor,” “non‐,” and “super” ILs. ILs with Walden plots close to the ideal line are classified as good ILs; those below the ideal line are poor ILs; and those far below are considered non‐ILs. Conversely, ILs whose plots lie above the ideal line are classified as super ILs. While this classification is largely qualitative, it provides a useful conceptual framework, analogous to the classification of dilute aqueous electrolyte solutions into “strong” and “weak” electrolyte solutions.

In this account, the classification of ILs based on Walden plots is further quantified using the molar conductivity calculated from the self‐diffusion coefficients of cations and anions measured by pulsed‐field gradient (PFG) NMR, instead of the KCl ideal line. We introduce the concept of “ionicity” of ILs as a quantitative alternative to deviation from the ideal Walden plot [13]. The ionicity of AILs is systematically examined and its physicochemical significance is discussed. Then, the ionicity and thermal stability of PILs prepared via proton transfer reactions from Brønsted acids to bases are analyzed in terms of the hydrogen‐bonding interactions between the protonated base and the resulting anion and the potential barrier for proton transfer reactions. The reactivity of active protons in PILs is exploited for proton‐conducting, nonaqueous electrolytes, and the potential of PILs as fuel cell electrolytes is also discussed.

## 2 Ionicity and Its Physicochemical Implications in Aprotic Ionic Liquids

Walden's rule (*Λη* = constant) for electrolytes composed of a monovalent cation and a monovalent anion can be derived from the Stokes–Einstein relation as follows [13, 14]

(1) $$\Lambda \eta =\alpha \frac{q}{6\pi }\left(\frac{1}{r_{+}}+\frac{1}{r_{-}}\right)$$

where *Λ* and *η* are the molar conductivity and viscosity of the electrolyte, *r*_+_ and *r*_−_ are the hydrodynamic radii of the cation and anion, respectively, and *q* is the ionic charge. The parameter *α* denotes the degree of dissociation; it approaches unity for strong aqueous electrolytes such as KCl(aq). The right‐hand‐side term of Equation (1) other than *α* may be treated as approximately constant if *r*_+_ and *r*_−_ do not vary markedly across ionic structures. However, because Walden plots of ionic liquids (ILs) often exhibit substantial dispersion (see Figure 1), this assumption can be a rough approximation. Under this approximation, the deviation of Walden plots from the ideal KCl line corresponds to *α*, i.e., when *α* is close to unity, such ILs are classified as good ILs, and when *α* is small, such ILs are classified as poor or non‐ILs. ILs whose Walden plots lie above the ideal line may involve special transport mechanisms, such as Grotthuss‐type proton hopping, that are less coupled to the bulk viscosity. MacFarlane and coworkers proposed an adjusted Walden plot that explicitly accounts for differences in ionic size [14].

We previously proposed a more quantitative assessment of transport properties in ionic liquids (ILs) by introducing self‐diffusion coefficients measured by pulsed‐field‐gradient NMR (PFG‐NMR). PFG‐NMR typically provides the cation diffusion coefficient (*D*_+_) via ^1^H NMR of cationic protons and the anion diffusion coefficient (*D*_−_) via ^19^F NMR. From *D*_+_ and *D*_−_, the molar conductivity expected from diffusivities can be estimated using the Nernst–Einstein relation, Equation (2), where *F*, *R*, and *T* have their conventional meanings

(2) $$\Lambda_{\mathrm{NMR}}=F^{2}\left(D_{+}+D_{-}\right)/RT$$

Here, $\Lambda_{\mathrm{NMR}}$ represents the molar conductivity assuming that the cation and anion self‐diffusion directly contributes to charge transport. By contrast, the molar conductivity associated with net charge transport, Equation (3), is obtained from the ionic conductivity (*σ*) measured by impedance spectroscopy

(3) $$\Lambda_{\mathrm{imp}}=\sigma /c$$

where *c* is the electrolyte concentration. We define “ionicity”, Equation (4), as the ratio of these two molar conductivities

(4) $$\mathrm{ionicity}=\Lambda_{\mathrm{imp}}/\Lambda_{\mathrm{NMR}}$$

Ionicity quantifies the extent to which ionic self‐diffusion (*Λ*_NMR_) contributes to the net charge transport (*Λ*_imp_). It generally ranges from zero to unity but can exceed unity when special transport mechanisms, such as structural (Grotthuss‐type) proton transfer, operate in ILs. Ionicity is also the inverse of the Haven ratio, a classical measure of correlated ionic motion in inorganic solid electrolytes.

**FIGURE 2:**
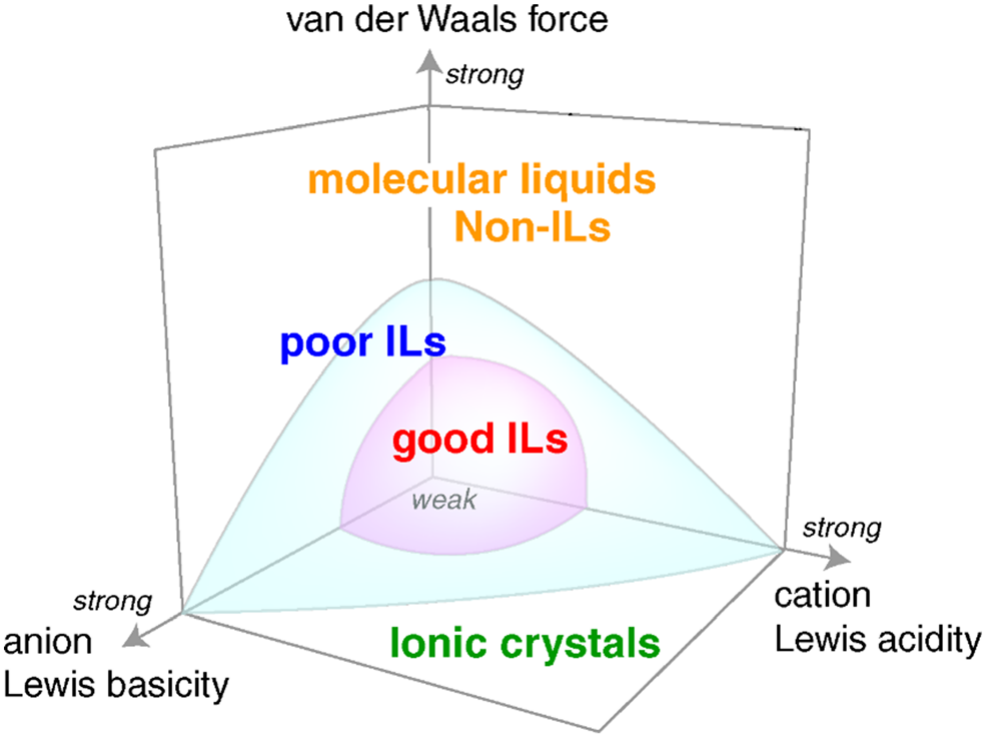
Conceptual scheme to express properties of ionic liquids (ILs) as functions of cationic Lewis acidity, anionic Lewis basicity, and van der Waals force (dispersion force). Good ILs mean ILs with high ionicity close to unity, while poor ILs mean ILs with low ionicity. Reproduced from Ref. [19] Copyright (2010) with permission from the Royal Society of Chemistry.

**FIGURE 3:**
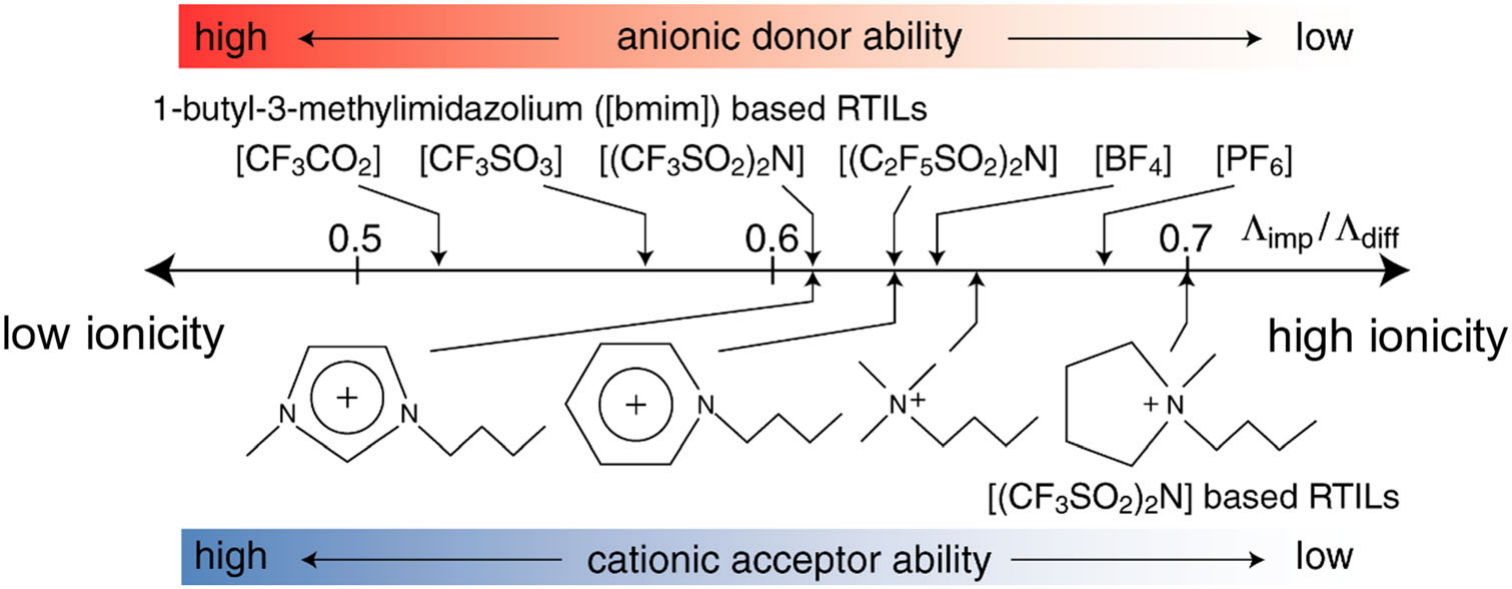
Ionicity of aprotic ILs depending on anionic structures for [bmim]‐based ILs and cationic structures for [(CF_3_SO_2_)_2_N]‐based ILs.

We systematically examined the ionicity of representative AILs [15, 16, 17, 18] and identified general trends (Figure 2) [19]. Three factors predominantly govern ionicity: (i) the Lewis basicity of the anion [15], (ii) the Lewis acidity of the cation [16], and (iii) the strength of van der Waals interactions (dispersion forces) [17]. When the cation Lewis acidity and anion Lewis basicity are both high while van der Waals forces are weak, the corresponding salts tend to form typical ionic crystals at ambient temperature (e.g., NaCl). In contrast, when cation Lewis acidity and anion Lewis basicity is both low and van der Waals interactions are weak, the salts form ILs with high ionicity—good ILs [18, 19]. As cation Lewis acidity, anion Lewis basicity, and van der Waals interactions increase, ionicity decreases and the ILs become poor ILs [18, 19]. When van der Waals interactions become very strong, for example, in ILs bearing long alkyl chains [17], the materials approach non‐IL behavior and ultimately resemble molecular liquids. Lewis acidity and basicity of cation and anion (representing charge distribution), respectively, dominantly determine Coulombic interaction between cation and anion. Anion Lewis basicity varies substantially with anion structure and strongly influences ionicity [15], as illustrated in Figure 3. On the cation side, variations in Lewis acidity are generally smaller; ILs based on aliphatic ammonium cations (e.g., pyrrolidinium, tetra‐alkylammonium) tend to show higher ionicity than those based on aromatic cations (e.g., pyridinium, imidazolium) [16]. The ring C–H protons in such aromatic cations are relatively acidic (i.e., proton‐donating) and interact preferentially with counter anions. Consequently, directionally specific interactions, equivalently, difference in ion–pair interaction energy depending on relative positions of cation and anion, also modulate ionicity [20].

As discussed in the first part of this section, the ionicity appears to be related to the dissociation/association of ions in ILs. In dilute weak electrolyte solutions, the following dissociation equilibrium, Equation (5), is established

(5) $$\underset{\mathrm{Dissociated}\;\mathrm{ions}}{M^{+}+X^{-}}⇌\underset{\mathrm{Ion}\;\mathrm{pairs}}{MX}$$

The dissociated ions contribute independently to the ionic conductivity, whereas neutral ion pairs do not. This assumption is valid under dilute conditions, where solute concentration is much lower than solvent concentration. In ILs, however, the situation differs fundamentally: ILs contain no neutral solvent and are composed entirely of ions. Consequently, contacted ions with other ions are major components in ILs and contribute to the ionic conductivity. In such cases, the lifetime of contacted ions and the exchange kinetics with surrounding ions (e.g., pair formation/dissociation or partner exchange) may determine the conductivity contribution.

The physicochemical meaning of ionicity remains debated [21, 22, 23]. In dense ionic fluids, including ionic liquids (ILs), ion transport must conserve local charge neutrality and momentum. In the absence of a molecular solvent, the momentum carried by one ion is balanced by counter‐motion of other ions, in contrast to dilute electrolyte solutions where the movement of solvent can keep the momentum preservation. Consequently, correlated ionic motion must be explicitly considered in dense ionic fluids.

Following Roling and Bedrov [24], ionic transport in dense ionic fluids can be formulated using Onsager reciprocal relations, Equation (6). The ionic conductivity is

(6) $$\sigma_{\mathrm{ion}}=\sigma_{++}+\sigma_{--}-2\sigma_{+-}$$

The Onsager coefficients *σ*_++_ and *σ*_−−_ can be decomposed into self and distinct parts, as shown by Equation (7)

(7) $$\sigma_{\mathrm{ion}}=\sigma_{+}^{\mathrm{self}}+\sigma_{++}^{\mathrm{distinct}}+\sigma_{‐}^{\mathrm{self}}+\sigma_{‐‐}^{\mathrm{distinct}}‐2\sigma_{+‐}$$

The self‐terms relate to the tracer diffusion coefficients *D*_+_ and *D*_−_ (e.g., PFG‐NMR diffusivities) via the Nernst–Einstein relations. The distinct terms, $\sigma_{++}^{\text{distinct}}$ and $\sigma_{--}^{\text{distinct}}$, quantify directional correlations among like‐charged ions, whereas $\sigma_{+-}$ captures directional correlations between oppositely charged ions. In an ideal electrolyte without ion–ion interactions (e.g., an aqueous solution at infinite dilution), such directional correlations vanish, as shown by Equation (8)

(8) $$\sigma_{++}^{\text{distinct}}=\sigma^{\text{distinct}}=\sigma_{+‐}=0$$

The Nernst–Einstein molar conductivity $\Lambda_{\mathrm{NMR}}$, obtained directly from *D*_+_ and *D*_−_, implicitly assumes this uncorrelated limit. In dense ionic fluids, especially ionic liquids, correlated motions must be included. Accordingly, ionicity can be expressed by Equation (9)

(9) $$\Lambda_{\mathrm{imp}}/\Lambda_{\mathrm{NMR}}=\sigma_{\mathrm{ion}}/(\sigma_{+}^{\text{self}}+\sigma_{‐}^{\mathrm{self}})$$

Negative correlated motion of cation and cation, or anion and anion ($\sigma_{++}^{\text{distinct}}<0$, $\sigma_{--}^{\text{distinct}}<0$), as in positional exchange of same‐charge ions, reduce ionicity. Likewise, positively correlated motion of cation and anion (*σ*_+−_ > 0), i.e., ionic motion to the same direction, lowers ionicity because it enters Equation (7) with a negative sign.

**FIGURE 4:**
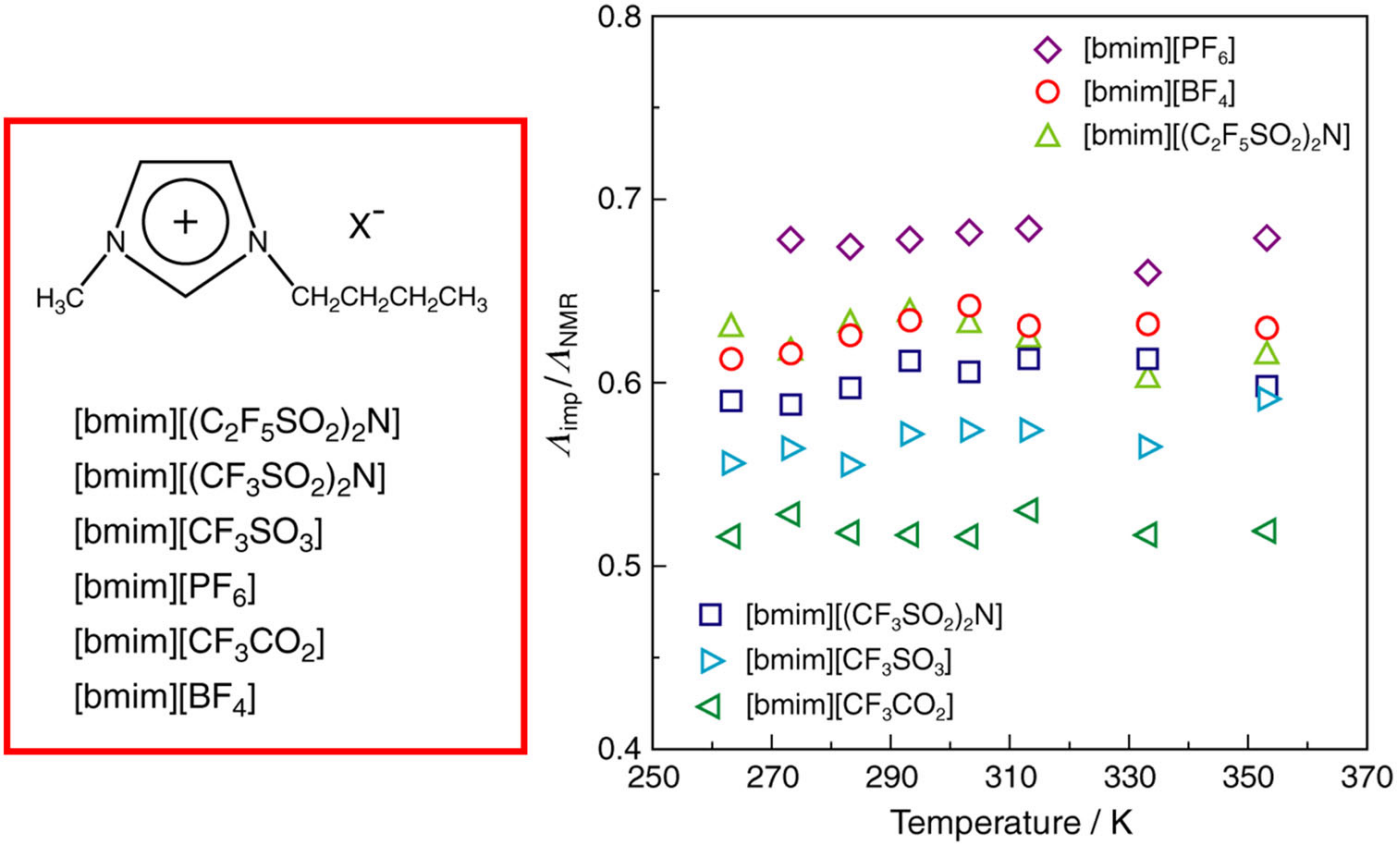
Temperature dependence of ionicity for imidazolium ([bmim])‐based ILs having different anions. Reproduced from Ref. [15] Copyright (2004) with permission from American Chemical Society.

Considering the correlated ionic motions discussed above, we re‐examined the ionicity trends of ILs in Figure 2 using more detailed experimental data. Figure 4 plots the ionicity of [bmim] (1‐butyl‐3‐methylimidazolium)‐based ILs with different anions as a function of temperature [15]. The ionicity is nearly temperature‐independent and decreases in the order: [bmim][PF_6_] > [bmim][BF_4_] ∼ [bmim][(C_2_F_5_SO_2_)_2_N] > [bmim][(CF_3_SO_2_)_2_N] > [bmim][CF_3_SO_3_] > [bmim][CF_3_CO_2_]. The Lewis basicity of these anions, estimated from the absorption maximum (λ_Cu_) of [Cu(acac)(tmen)][BPh_4_] (acac = acetylacetonate; tmen = tetramethylethylenediamine) and from the ^1^H NMR chemical shift of the C_2_–H in [bmim], follows the sequence [15, 18]: [bmim][PF_6_] < [bmim][BF_4_] ∼ [bmim][(C_2_F_5_SO_2_)_2_N] < [bmim][(CF_3_SO_2_)_2_N] < [bmim][CF_3_SO_3_] < [bmim][CF_3_CO_2_]. Thus, the ionicity order is the inverse of the Lewis basicity order. Ab initio calculations (MP2/6‐311G** level) further show that the formation energies (kcal mol^−1^) of the most stable [bmim] ion pairs follow: [PF_6_] (−76.1) < [(CF_3_SO_2_)_2_N] (−76.6) < [CF_3_SO_3_] (−81.3) < [BF_4_] (−83.1) < [CF_3_CO_2_] (−87.8). It is also reported that ion‐pair formation energies vary little among quaternized cations (imidazolium, pyridinium, ammonium, pyrrolidinium) [20]. Although the formation energy of [emim][BF_4_] is slightly higher than expected from the ionicity trend and the anion Lewis basicity, the overall ionicity order is broadly consistent with the cation–anion interaction energies. The decrease in ionicity with increasing ion‐pair formation energy can be rationalized by a positive *σ*_+−_ term, i.e., correlated same‐direction motion of cations and anions. The associated momentum may be sustained by oppositely directed, same‐directional correlations among neighboring cations and anions.

**FIGURE 5:**
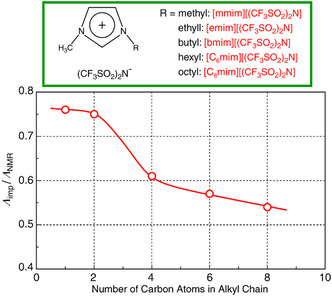
Ionicity at 30°C for imidazolium‐based ILs having different alkyl chain lengths. The anion is common ([(CF_3_SO_2_)_2_N]). Lines in the figure are just a guide for eyes. Reproduced from Ref. [17] Copyright (2005) with permission from American Chemical Society.

Figure 5 shows the ionicity of the [C_*n*_mim][(CF_3_SO_2_)_2_N] ([C_*n*_mim] = 1‐alkyl (CH_3_(CH_2_)_*n*−1_)‐3‐methylimidazolium) as a function of the alkyl chain length *n* [17]. Notably, ionicity decreases with increasing *n*, with a particularly sharp drop from *n* = 3 to *n* = 4. Numerous studies indicate that ionic liquids are not homogeneous but instead exhibit a “liquid‐mosaic” nanostructure, in which polar and nonpolar components segregate on the nanometer scale [25, 26]. As the alkyl chain on the cation lengthens, the charged headgroups and anions tend to distribute as uniformly as possible, while the neutral tails aggregate into nonpolar domains. This behavior reflects a balance between electrostatic interactions among the charged species and collective short‐range interactions among the neutral tails, which minimize disruption of the electrostatic network.

**FIGURE 6:**
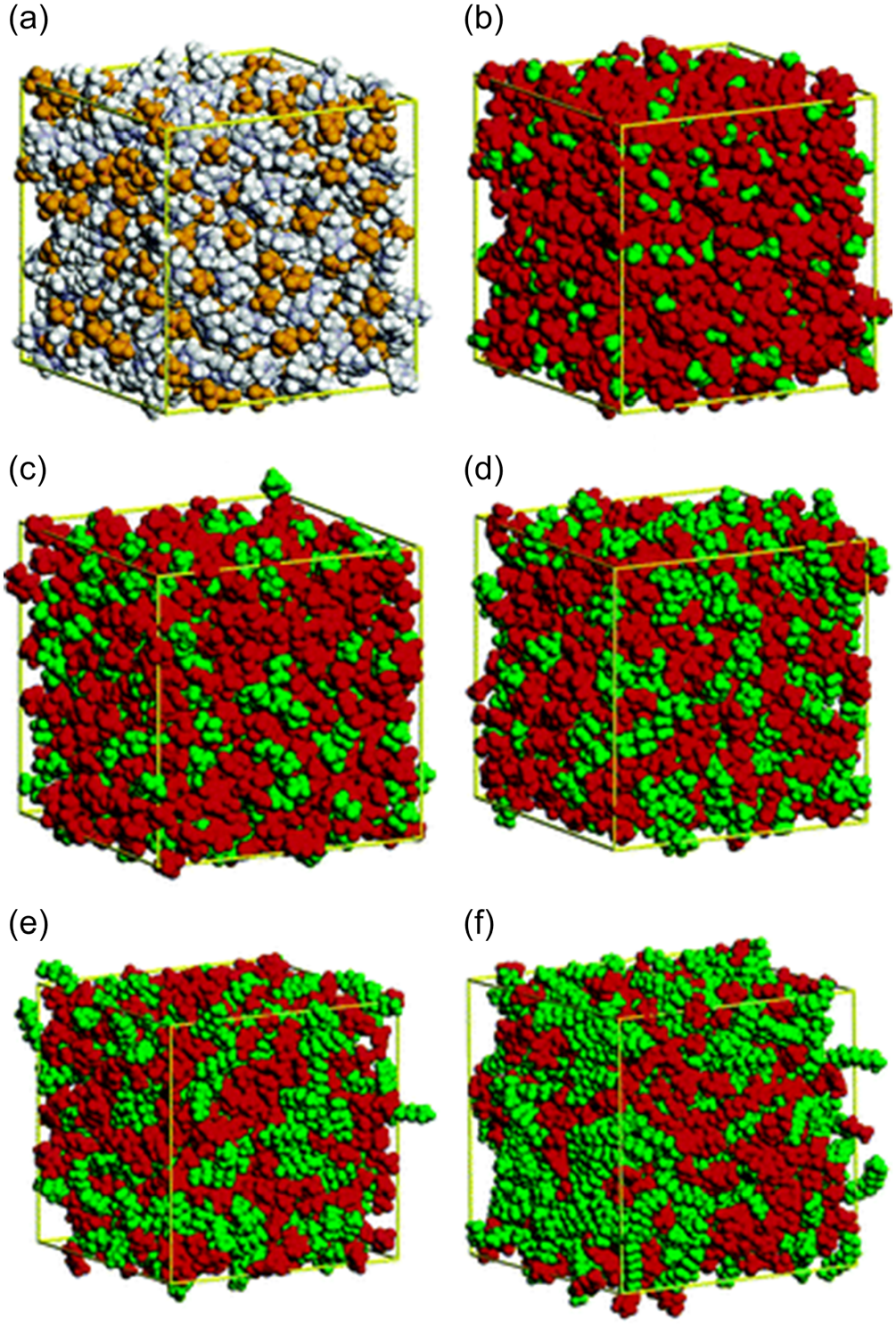
Snapshots of simulation boxes containing 700 ions of [C_*n*_mim][PF_6_]. The application of a coloring code enables clear identification of the charged (red) and nonpolar (green) domains that form in ionic liquids. The lengths of the box sides are given: (a) [C_2_mim][PF_6_] (CPK coloring); (b) [C_2_mim][PF_6_]; (c) [C_4_mim][PF_6_]; (d) [C_6_mim][PF_6_]; (e) [C_8_mim][PF_6_]; (f) [C_12_mim][PF_6_]. Reproduced from Ref. [25] Copyright (2006) with permission from the American Chemical Society.

In [C_*n*_mim][PF_6_] or [C_*n*_mim][(CF_3_SO_2_)_2_N] with *n* ≥ 4, aggregation of the alkyl chains into nonpolar domains is observed (Figure 6) [25]. These domains percolate around a three‐ dimensional network of ionic channels formed by the anions and the imidazolium ring regions of the cations. As *n* increases, the nonpolar domains grow and become more connected, swelling and constraining the ionic network. Such nano‐segregation helps rationalize several experimentally observed properties of ionic liquids [26].

The ionicity trend in Figure 5 [17] can be understood if anticorrelated like‐ion motions become more pronounced as *n* increases, i.e., $\sigma_{++}^{\text{distinct}}<0$. In a phase‐segregated morphology, momentum associated with the motion of a large cation can be compensated by the anticorrelated motion of neighboring cations, thereby reducing the net ionic conductivity. The emergence and strengthening of these negative distinct terms provide a natural explanation for the overall decrease in ionicity with chain length, and particularly for the abrupt drop between *n* = 3 and *n* = 4, where nano‐segregation becomes prominent.

## 3 Ionicity and Thermal Stability of Protic Ionic Liquids

**SCHEME 1:**
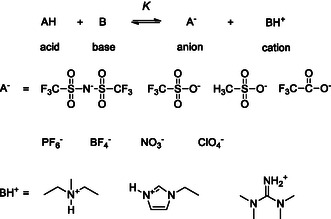
Acid–base equilibrium in protic ionic liquids (PILs). A^−^ and BH^+^ are representative anions and cations comprising PILs, respectively. Reproduced from Ref. [28] Copyright (2017) with permission from the American Chemical Society.

PILs are ionic liquids that contain at least one labile (active) proton [9, 10]. They are typically synthesized via an acid–base neutralization (proton‐transfer) reaction between a Brønsted acid and a Brønsted base (Scheme 1) [27, 28, 29]. For Brønsted acid/base‐derived PILs, the equilibrium shifts toward ionic species as Δ*p* *K*_a_ increases, where

(10) $$\Delta pK_{a}=p{K_{a}}^{\mathrm{BH}+/B}-p{K_{a}}^{\mathrm{HA}/A-}=\log K$$

because the Gibbs energy change for neutralization (−*RT* ln *K*) becomes more negative with increasing Δ*p* *K*_a_. This treatment assumes that hydration energies in aqueous media are negligible compared with the large Gibbs energy change of neutralization. Using potentiometric titration, Kanzaki et al. determined formation constants (*K*) for several PILs; for ethylammonium nitrate, the measured *K* is lower than that derived from Δ*p* *K*_a_ by 2 orders of magnitude [30]. Nevertheless, the working assumption is supported by the observed linear correlation between of logarithm of *K* in the bulk PIL and that in water [31]. Dai et al. subsequently reported thermally stable PILs prepared from superacids and organic superbases such as phosphazenes and guanidines (Scheme 1) [32].

**FIGURE 7:**
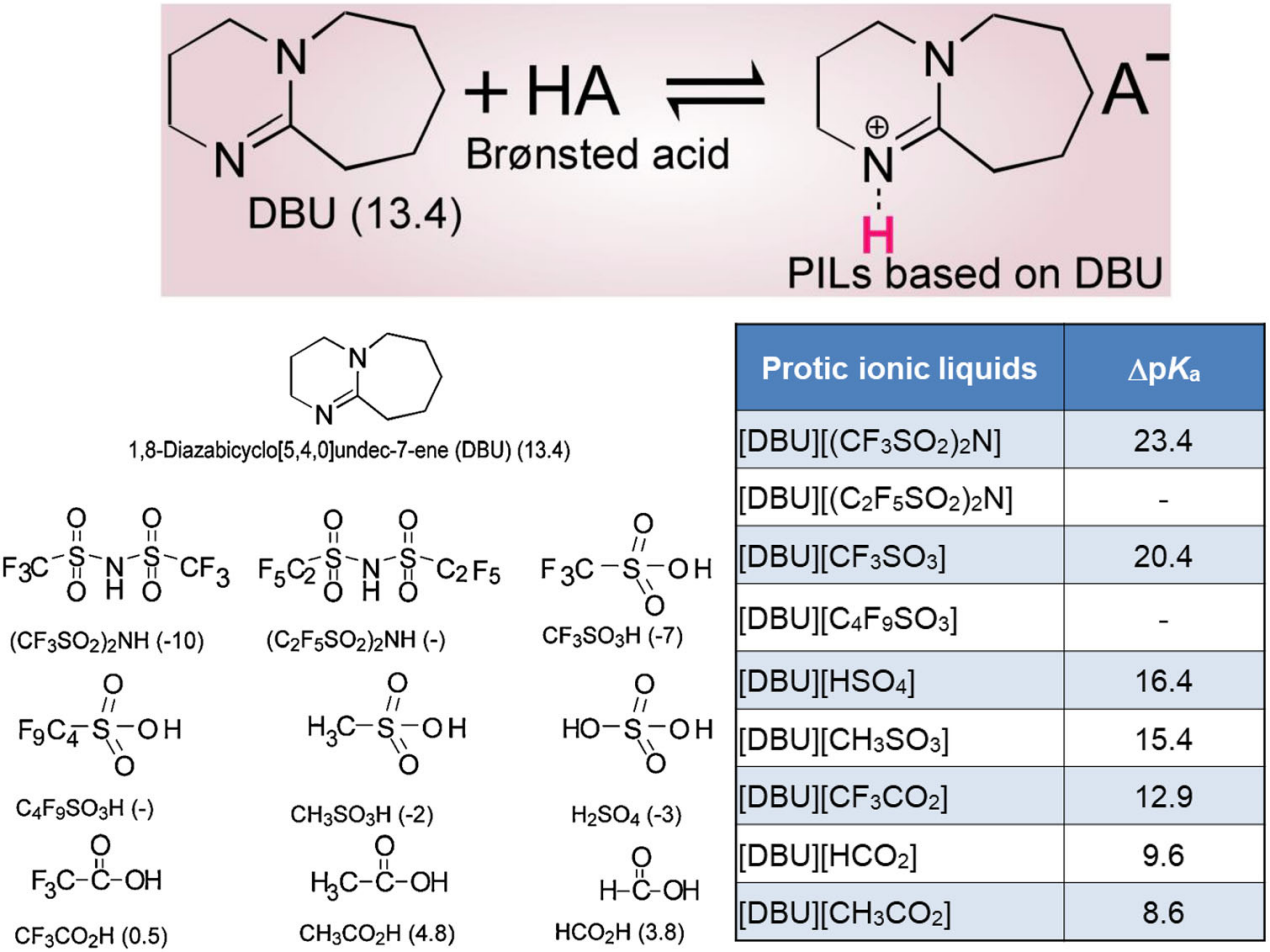
Formation of an organic superbase (DBU)‐based protic ILs with a variety of acids (number in parenthesis indicates *p* *K*_a_) and Δ*p* *K*_a_ of resulting protic ILs.

**FIGURE 8:**
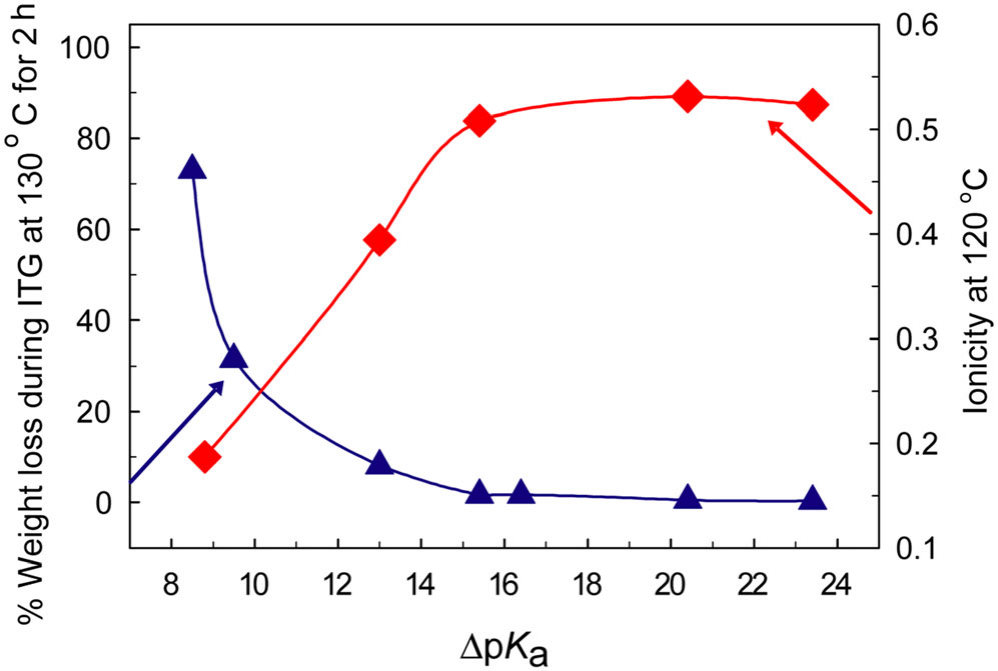
Weight loss after heating at 130°C for 2 h under an N_2_ atmosphere and ionicity at 120°C of DBU‐based protic ILs as a function of Δ*p* *K*_a_. Lines in the figure are just a guide for eyes. Reproduced from Ref. [33] Copyright (2012) with permission from the Royal Society of Chemistry.

We investigated the thermal stability and ionicity of PILs based on 1,8‐diazabicyclo [5.4.0]undec‐7‐ene (DBU) and a range of Brønsted acids (Figure 7) [33]. Because DBU is an organic superbase, a wide range of Δ*p* *K*_a_ values can be accessed by selecting appropriate acids. All equimolar DBU–acid mixtures formed homogeneous liquids at room temperature. Figure 8 summarizes the thermal stability (isothermal mass loss at 130°C for 2 h) and ionicity of these liquids at 120°C [33]. Notably, as Δ*p* *K*_a_ increases, mass loss decreases sharply while ionicity increases. When Δ*p* *K*_a_ exceeds ∼15, mass loss becomes negligible and the ionicity rises to slightly above 0.5. Although the ionic conductivities are somewhat lower than those of typical AILs, they reach ∼10^−3^ S cm^−1^ at sufficiently large Δ*p* *K*_a_.

**FIGURE 9:**
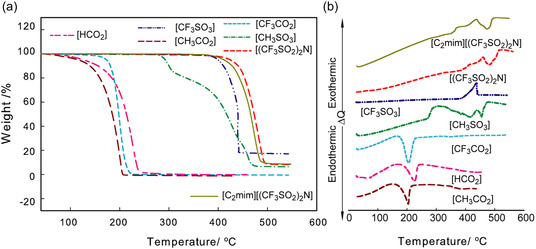
(a) Thermogravimetric (TG) and (b) differential scanning calorimetry (DSC) traces of DBU‐based protic ILs. Reproduced from Ref. [33] Copyright (2012) with permission from the Royal Society of Chemistry.

Figure 9a,b shows thermogravimetric (TG) and differential scanning calorimetry (DSC) traces, respectively [33]. For liquids with Δ*p* *K*_a_ < 15, the TG curves (Figure 9a) indicate complete mass loss below 300°C, whereas markedly enhanced thermal stability is observed for Δ*p* *K*_a_ > 15. The thermal stability of [DBU][(CF_3_SO_2_)_2_N] is slightly higher than that of [C_2_mim][(CF_3_SO_2_)_2_N], which is known as one of the most thermally robust AILs [17]. The DSC data (Figure 9b) further reveal that the mass‐loss process is endothermic for Δ*p* *K*_a_ < 15 but becomes exothermic for Δ*p* *K*_a_ > 15; a similar exothermic decomposition is observed for the thermally stable AIL, [C_2_mim][(CF_3_SO_2_)_2_N].

A simple explanation for the poor thermal stability at Δ*p* *K*_a_ < 15 would be an insufficient formation constant (*K*) for the PILs, i.e., a significant fraction of neutral species persists at low Δ*p* *K*_a_. Such an explanation is reasonable when Δ*p* *K*_a_ is very small or negative. For example, aqueous NH_3_ is basic via NH_3_ + H_2_O ⇌ NH_4_^+^ + OH^−^, where water serves as a Brønsted acid toward NH_3_; however, the Δ*p* *K*_a_ of this combination is −6.45, and NH_4_OH cannot be isolated due to the evaporation of NH_3_ and H_2_O and the subsequent equilibrium shift toward nonionic species. The DBU‐based systems considered here differ from this NH_4_OH case.

**FIGURE 10:**
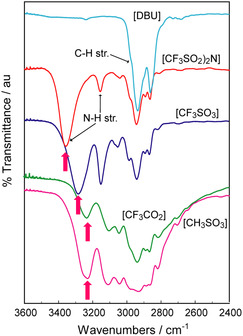
FT‐IR spectra for DBU‐based protic ILs at room temperature. Reproduced from Ref. [34] Copyright (2011) with permission from the Royal Society of Chemistry.

**FIGURE 11:**
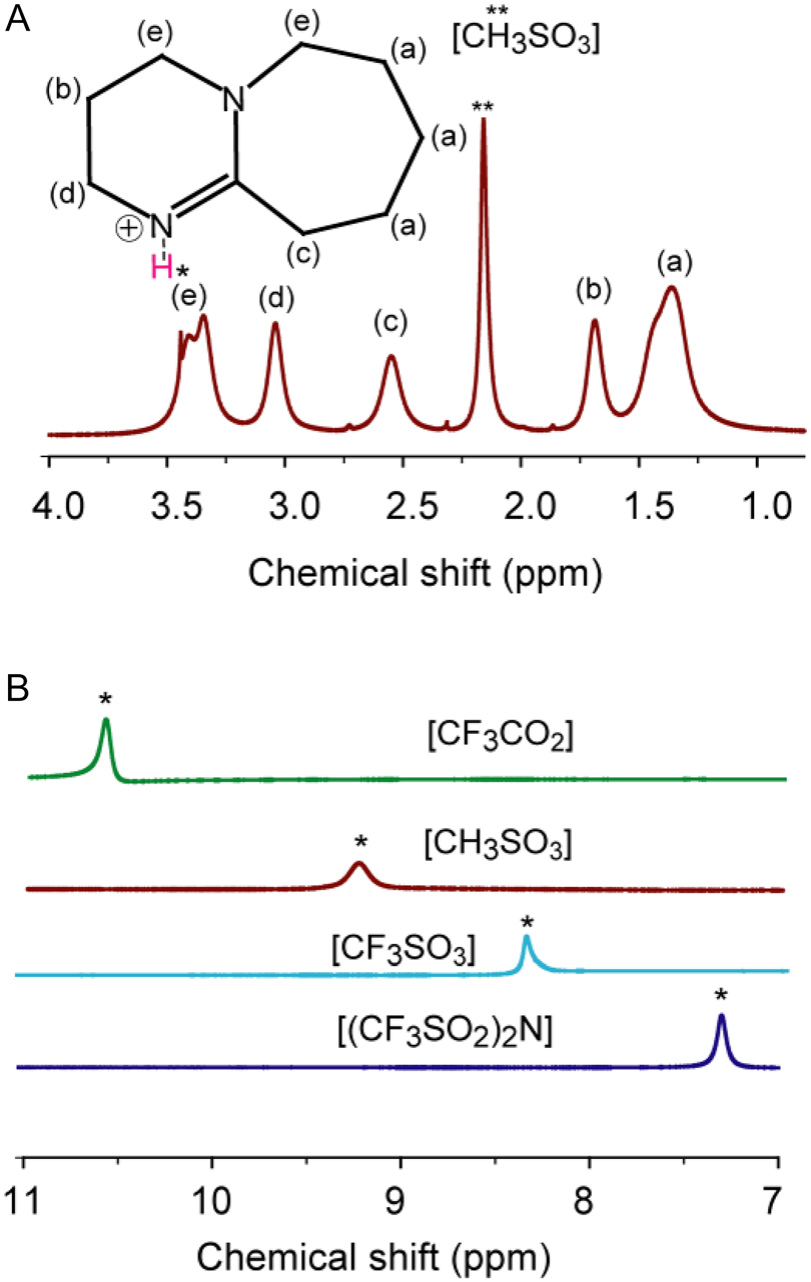
^1^H NMR spectra of [DBU][CH_3_SO_3_] in upper field region (A) and N‐H protons of DBU‐based protic ILs with different anions (B). Reproduced from Ref. [34] Copyright (2011) with permission from the Royal Society of Chemistry.

Figure 10 shows FT‐IR spectra of the equimolar mixtures [34]. Relative to neat DBU, the NH stretching band (*v*_NH_) of protonated DBU is clearly observed in most mixtures. In the case of [DBU][CH_3_CO_2_], the *v*_NH_ becomes broad and obscure. Importantly, the *v*_NH_ wavenumber increases with Δ*p* *K*_a_, for example, 3231 cm^−1^ for [DBU][CF_3_CO_2_] (Δ*p* *K*_a_ = 12.9) and 3354 cm^−1^ for [DBU][(CF_3_SO_2_)_2_N] (Δ*p* *K*_a_ = 23.4). This trend reflects differences in hydrogen (H)‐bond strength between the NH of protonated DBU and the conjugate base anions: lower Δ*p* *K*_a_ corresponds to stronger H‐bonding. ^1^H NMR spectra (Figure 11) corroborate both the protonation of DBU and the variation in H‐bond interactions: the chemical shift (*δ*) of the NH proton depends sensitively on Δ*p* *K*_a_ (B), whereas the other proton resonances remain essentially unchanged (A) [34]. The NH signal (*δ*) shifts downfield in the order of anions: [(CF_3_SO_2_)_2_N] (Δ*p* *K*_a_ = 23.4) < [CF_3_SO_3_] (Δ*p* *K*_a_ = 20.4) < [CH_3_SO_3_] (Δ*p* *K*_a_ = 15.4) < [CF_3_CO_2_] (Δ*p* *K*_a_ = 12.9). Taken together the FT‐IR and ^1^H NMR results and the generally large Δ*p* *K*_a_ values, we conclude that proton transfer from the acids to DBU proceeds to give PILs with negligible neutral species.

**FIGURE 12:**
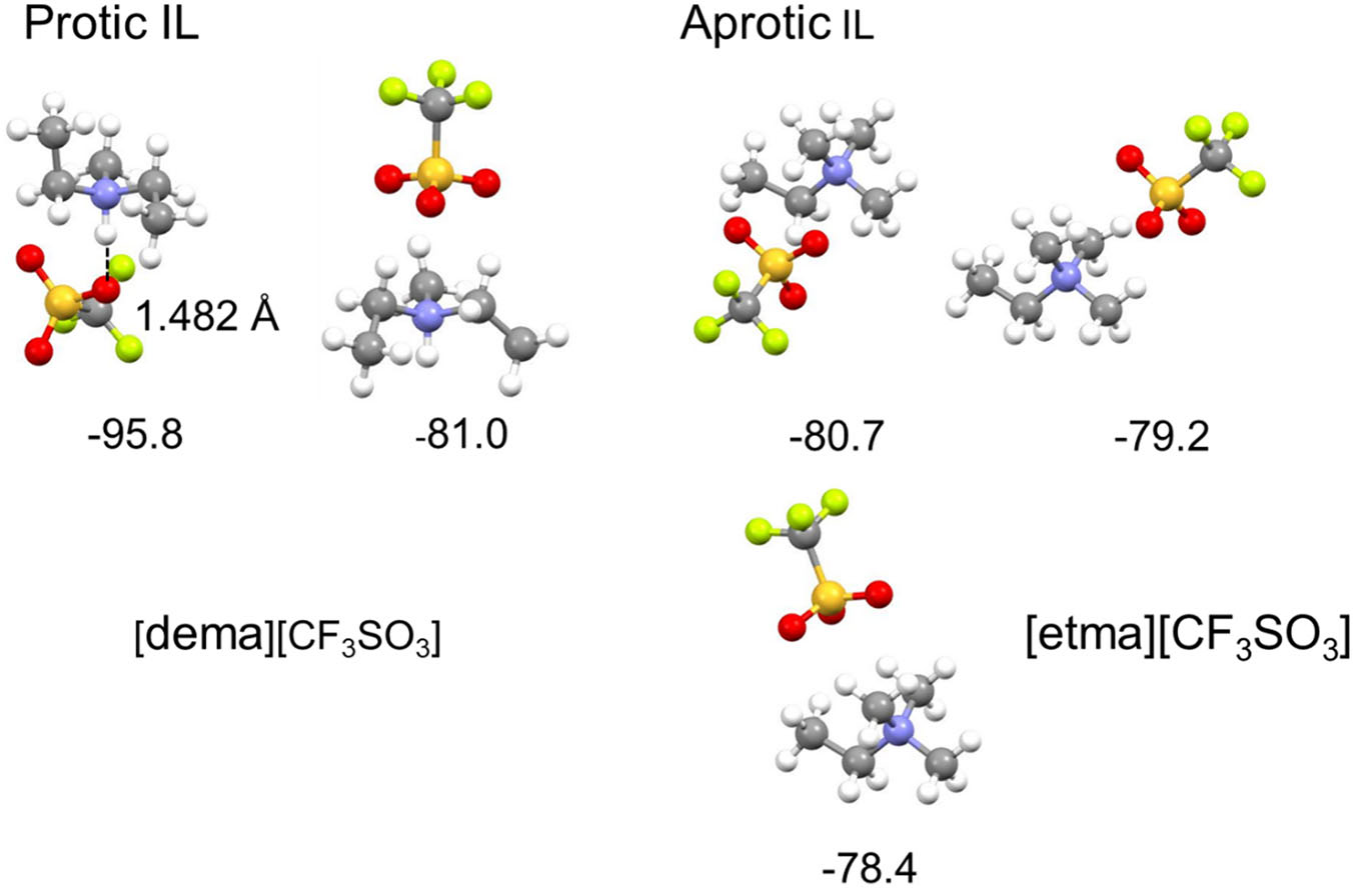
Stable ion‐pair structures and formation energies (MP2/6‐311G^∗∗^ level ab initio calculations, formation energy: kcal mol^−1^) for structurally isomeric protic IL ([dema][CF_3_SO_3_]) and aprotic IL ([etma][CF_3_SO_3_]).

Regarding the ionicity of these PILs, the values are relatively low compared with typical AILs (Figures 4 and 5). Figure 12 presents stable ion‐pair structures and formation energies (kcal mol^−1^) for two structural isomers, one PIL and one AIL, namely [dema][CF_3_SO_3_] and [etma][CF_3_SO_3_], respectively, where [dema] and [etma] denote diethylmethylammonium and ethyltrimethylammonium [35]. For the AIL [etma][CF_3_SO_3_], the formation energy is approximately −80 kcal mol^−1^ and is essentially independent of the relative cation–anion orientation; in other words, the interaction is only weakly directional. By contrast, in the PIL [dema][CF_3_SO_3_] the formation energy depends strongly on the relative positioning of the ions. When the anion is located opposite the cation's NH proton, the formation energy is ca. −80 kcal mol^−1^, comparable to that of [etma][CF_3_SO_3_]. However, when the anion approaches the vicinity of the NH proton, the formation energy deepens to ca. −95 kcal mol^−1^, indicating that H‐bonding significantly stabilizes the ion pair.

**FIGURE 13:**
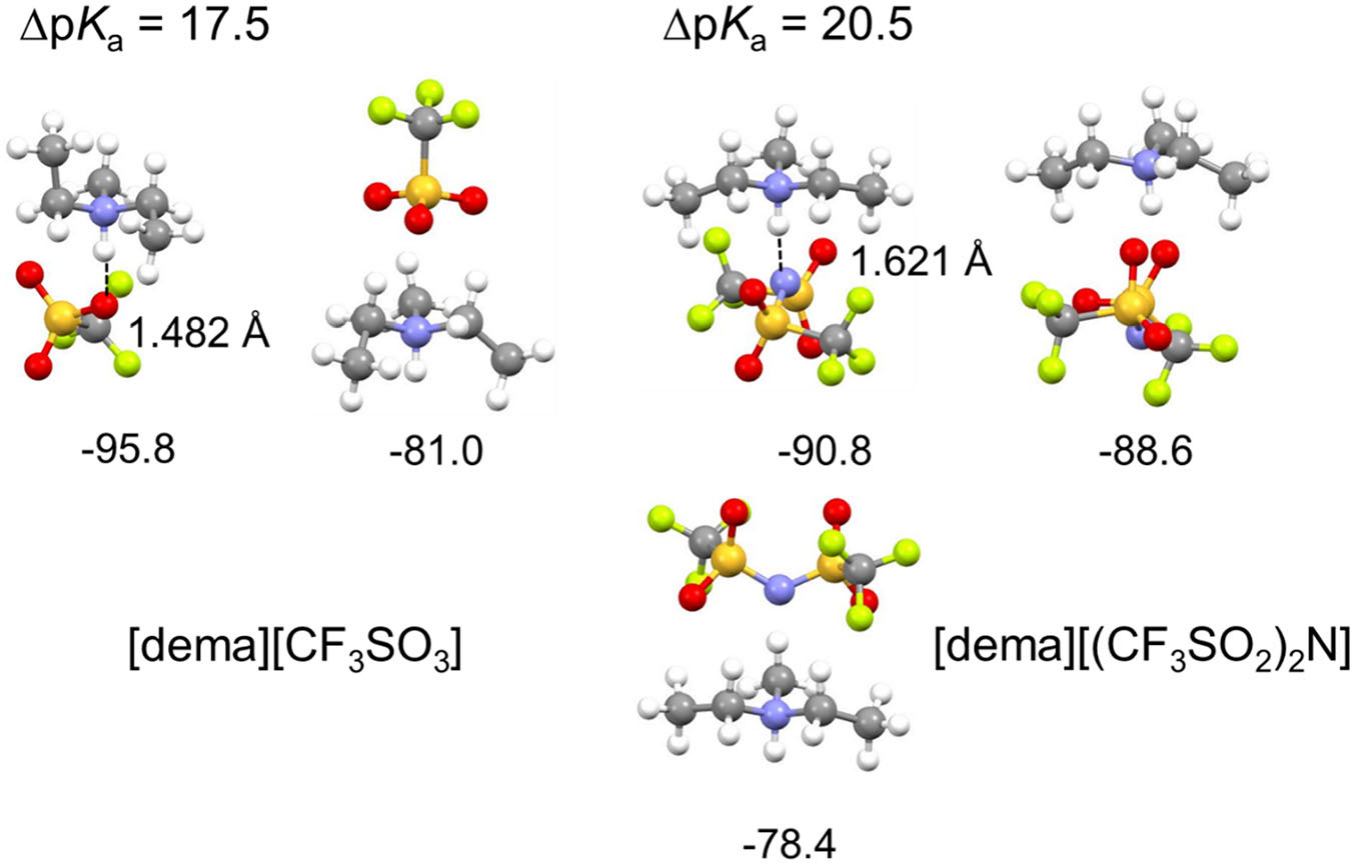
Stable ion‐pair structures and formation energies (MP2/6‐311G^∗∗^ level ab initio calculations, formation energy: kcal mol^−1^) for [dema][CF_3_SO_3_] (Δ*p* *K*_a_ = 17.5) and [dema][(CF_3_SO_2_)_2_N] (Δ*p* *K*_a_ = 20.5). The closest distances between NH proton and O atom ([dema][CF_3_SO_3_]) and N atom ([dema][(CF_3_SO_2_)_2_N]) are indicated.

Figure 13 shows stable ion‐pair structures and formation energies (kcal mol^−1^) for [dema][CF_3_SO_3_] (Δ*p* *K*_a_ = 17.5) and [dema][(CF_3_SO_2_)_2_N] (Δ*p* *K*_a_ = 20.5) [35]. Again, these PILs exhibit pronounced directionality in terms of the ion‐pair formation energies, and for the most stable ion pairs, the shortest cation–anion distance (N−H···O or N−H···N) is smaller for [dema][CF_3_SO_3_]. Table 1 summarizes the N−H distance (on the [dema] side) and the H···O (H···N) distance (on the acid side) for the most stable ion pairs of [dema]‐based protic ILs, calculated at the MP2/6‐311G** level. It is interesting to note that, as Δ*p* *K*_a_ decreases, the N−H distance increases, whereas the H···O (H···N) distance decreases. This trend supports the conclusion that the H‐bonding interaction strengthens as Δ*p* *K*_a_ decreases. For the [dema]‐based protic ILs, where the *p* *K*_a_ of [dema] is 10.35, the relative magnitudes of these two distances are inverted at [dema][CF_3_CO_2_] (Δ*p* *K*_a_ = 9.85). For protic ILs with larger Δ*p* *K*_a_ values (i.e., up to [dema][CF_3_CO_2_]), the N−H (covalent) bond is shorter than the H···O (H···N) (H‐bond) distance, whereas for Δ*p* *K*_a_ ≤ 9.85, the N−H bond becomes longer than the H···O (H···N) distance. This indicates that proton‐transfer reactions are unfavorable for the low‐Δ*p* *K*_a_‐protic ILs at the MP2/6‐311G** level of ab initio calculation. It should also be noted that the *p* *K*_a_ of [dema] is lower than that of [DBU] by 3, so proton transfer would be more facilitated in the [DBU]‐based protic ILs.

**TABLE 1:** N−H (on the [dema] side) and H···O (H···N) (on the acid side) distances for the most stable ion‐pairs (MP2/6‐311G** level) for [dema]‐based protic ionic liquids with different Δ*p* *K*_a_.

Protic ionic liquid	Δ*p* *K*_a_	N‐H distance/Å	H···O (H···N) distance/Å
[dema][(CF_3_SO_2_)_2_N]	20.35	1.072	1.621
[dema][CF_3_SO_3_]	17.35	1.086	1.482
[dema][HSO_4_]	13.35	1.101	1.442
[dema][CH_3_SO_3_]	12.35	1.118	1.405
[dema][CF_3_CO_2_]	9.85	1.533	1.053
[dema][HCO_2_]	6.55	1.653	1.020
[dema][CH_3_CO_2_]	5.55	1.669	1.015

The low ionicity of PILs in Figure 8 can thus be rationalized if the positive cross‐correlation term *σ*_+−_ becomes predominant as H‐bonding between the cation and anion becomes stronger (i.e., with decreasing Δ*p* *K*_a_). Even as Δ*p* *K*_a_ increases, the ionicity tends to saturate at ∼0.5 (Figure 8), remaining below that of typical AILs. Notably, H‐bonding interactions persist even at high‐Δ*p* *K*_a_ PILs (Figure 13), contributing to a positive *σ*_+−_.

**FIGURE 14:**
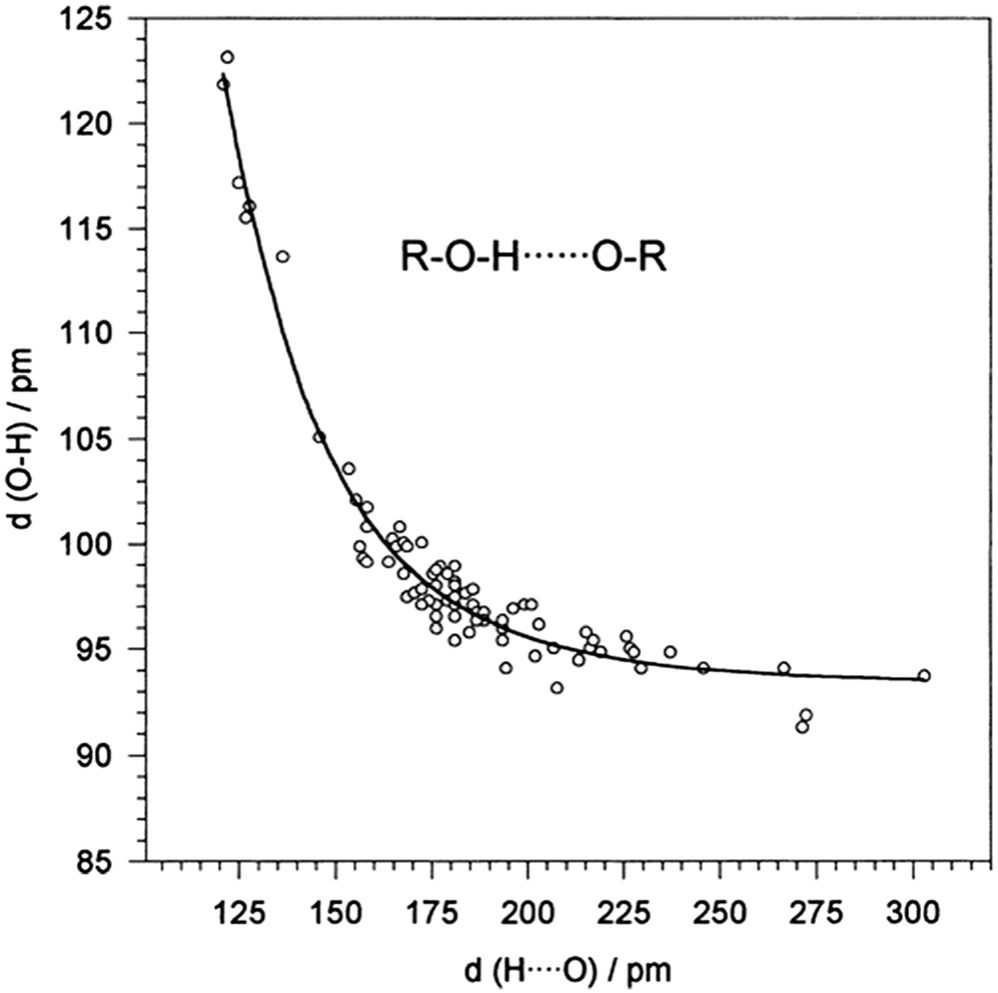
Bond distance relationship for hydrogen bonds of the type R−O−H···O−R in a variety of compounds. Reproduced from Ref. [36] Copyright (1996) with permission from the American Chemical Society.

**FIGURE 15:**
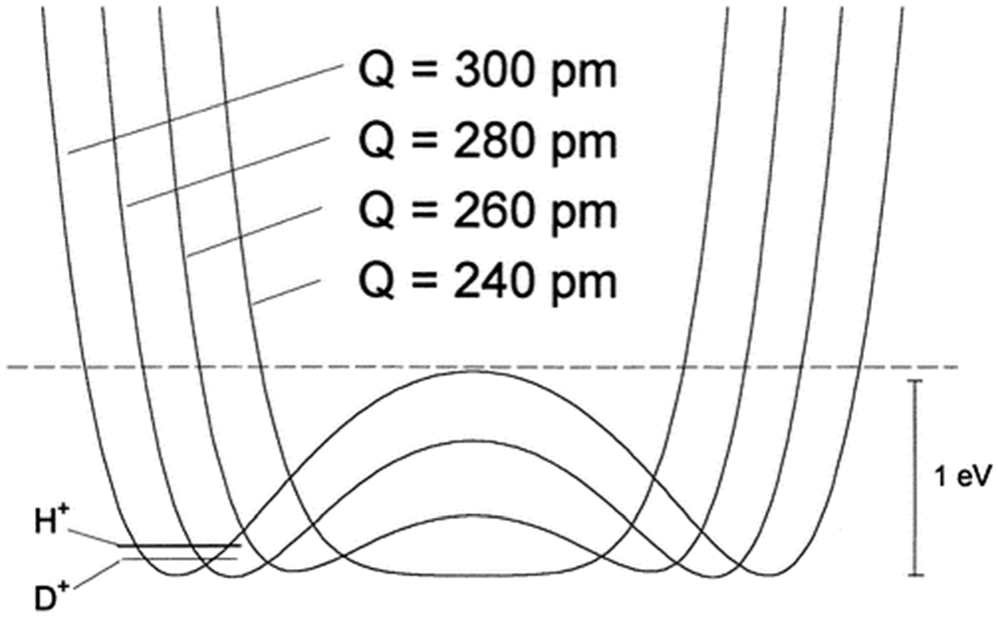
Semiempirical potential for proton transfer along hydrogen bonds of symmetrical configurations of the type R−O−H···O−R for different oxygen separations *Q* and full relaxation of the environment. For one potential (*Q* = 300 pm) the H^+^ and D^+^ vibrational ground states and the transition state for proton (deuteron) transfer are indicated. Reproduced from Ref. [36] Copyright (1996) with permission from the American Chemical Society.

A plausible reason for the poor thermal stability of PILs with relatively low Δ*p* *K*_a_ (Figure 8) is considered here. A classic ab initio study on diverse model systems (intra‐ and intermolecular; ionic and neutral H‐bonds) shows that as an H‐bond becomes stronger—shorter and more linear—the potential‐energy barrier for proton transfer decreases and can even vanish, merging into a single‐well potential and thereby reducing the barrier for back proton transfer. Figure 14 illustrates correlated changes in H‐bond distance and covalent bond length for a series of R–O–H···O–R hydrogen bonds from crystallographic data [36]. Across many compounds, shorter (stronger) H‐bonds are accompanied by longer (weaker) O–H covalent bonds. Figure 15 shows how the proton‐transfer barrier varies with the O···O separation (*d*_O−O_ = *Q*) in R–O–H···O–R motifs, where *Q* is a metric of the strength of H‐bonds. As *d*_O−O_ decreases (stronger H‐bonding), the barrier is lowered and the potential surface tends to flatten or become single‐welled [36]. Given the generality of the trends in Figures 14 and 15, the relatively low thermal stability and endothermic mass loss of low‐Δ*p* *K*_a_ PILs (Figures 8 and 9) can plausibly be attributed to facile reverse proton‐transfer reactions and evaporation of the resulting neutral species (DBU and the corresponding acids).

## 4 Electrochemical Reactivity of Protic Ionic Liquids

A key chemical distinction between PILs and AILs is the presence of labile (acidic) protons in the former and their absence in the latter, alongside differences in thermal stability and ionic conductivity. The acidic protons in PILs can be readily reduced, evolving hydrogen gas. Motivated by the work of Kreuer and coworkers [36, 37], we hypothesized that PILs could serve as proton‐conducting electrolytes for fuel cells under nonhumidified conditions. In conventional proton conductors (i.e., acidic solutions and perfluorosulfonic acid membranes such as Nafion), water acts as a Brønsted base for acidic protons, as shown by Equation (11)

(11) $$\mathrm{AH}+H_{2}O\to A^{-}+H_{3}O^{+}$$

Generated hydronium cation (H_3_O^+^) transports protons via either the vehicular or Grotthuss mechanism. In PILs, protons are accepted by Brønsted bases, typically amines as Equation (12)

(12) $$\mathrm{AH}+{\mathrm{NR}}_{3}\to A^{-}+\mathrm{HN}{R_{3}}^{+}$$

If the PIL exhibits high conductivity and the conjugate acid HNR_3_^+^ is electroactive toward the relevant fuel‐cell reactions, the PIL can function as a proton conductor under nonhumidified conditions at temperatures above 100°C. We initiated studies of PILs as anhydrous proton conductors using mixtures of imidazole (Im) and H[(CF_3_SO_2_)_2_N [38, 39]. These mixtures form liquids over broad composition and temperature ranges. The ionic conductivity exhibits a maximum under Im‐rich conditions, where proton transport occurs through both migration of [HIm] (vehicular mechanism) and proton hopping among H‐bonded imidazole molecules (Grotthuss mechanism). However, at strongly base‐ or acid‐rich compositions, the thermal stability of these liquids is insufficient for fuel‐cell applications [38, 39], even when a more thermally stable base, benzimidazole, is used [40]. Similarly, low‐Δ*p* *K*_a_ PILs readily release and accept protons through strong H‐bonds, which enhance Grotthuss‐type proton conduction [41]; however, their thermal stability is poor, as mentioned in Section 3.

**FIGURE 16:**
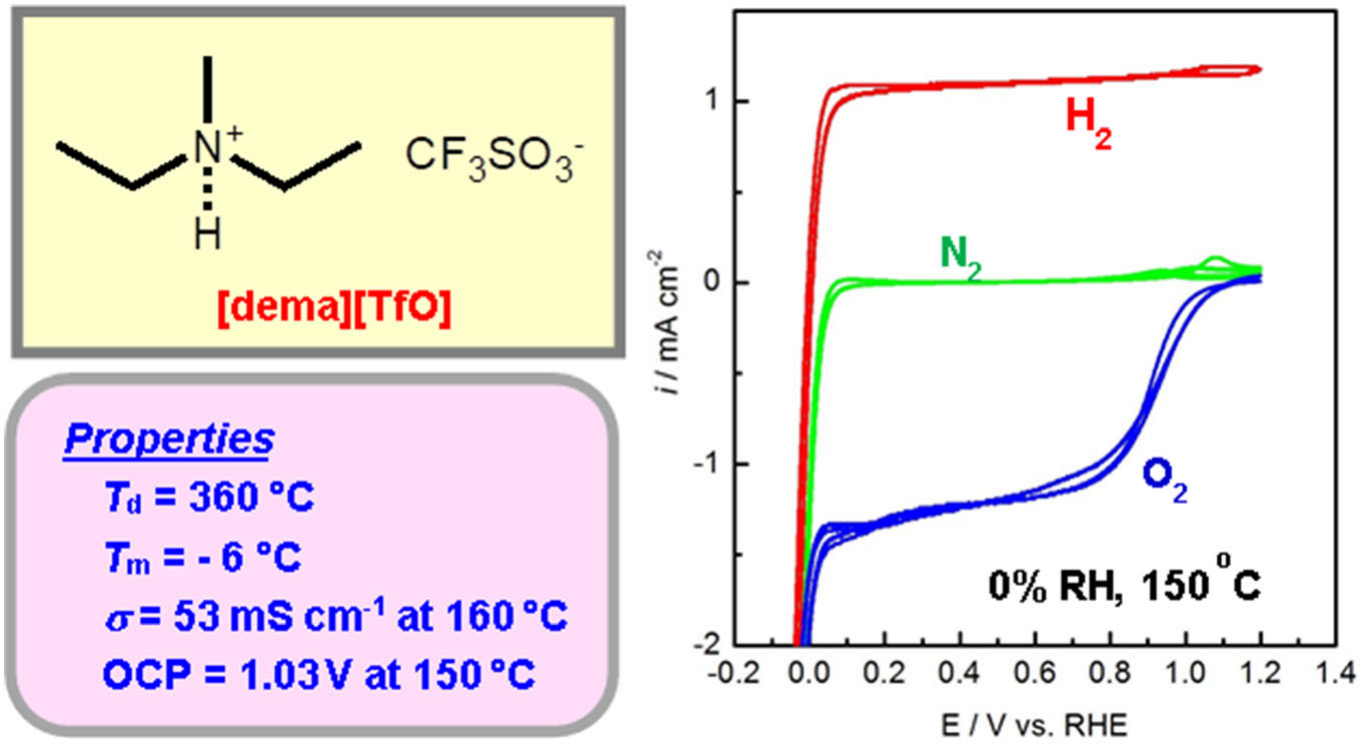
Bulk and electrochemical properties of [dema][CF_3_SO_3_]. The cyclic voltammograms (right) are recorded at 150°C under N_2_, H_2_, O_2_ bubbling conditions at a Pt wire working electrode equipped with a platinized Pt wire counter electrode. A reference electrode, RHE, is a Pt wire in H_2_ bubbling conditions, placed close to the working electrode through a Luggin capillary. Reproduced from Ref. [43] Copyright (2016) with permission from The Electrochemical Society of Japan.

After screening several different PILs, [dema][CF_3_SO_3_] was identified as a thermally stable, highly conductive, and electrochemically active PIL suitable for use as an anhydrous proton conductor [42]. Specifically, [dema][CF_3_SO_3_] has a melting temperature *T*_m_ = −6°C, a decomposition temperature *T*_d_ = 360°C, and an ionic conductivity *σ* = 53 mS cm^−1^ at 160°C. Figure 16 shows steady‐state cyclic voltammograms at a Pt electrode in [dema][CF_3_SO_3_] under different atmospheres at 150°C [43]. Under inert (N_2_)‐bubbling atmosphere, [dema][CF_3_SO_3_] undergoes reduction at potentials below 0 V vs. the reversible hydrogen electrode (RHE), which can be ascribed to hydrogen evolution from the [dema] cation. Under O_2_‐bubbling atmosphere, a reduction current starts to flow at 1.03 V vs. RHE. Under H_2_‐bubbling atmosphere, the steady‐state voltammetry shows cathodic and anodic currents below and above 0 V vs. RHE, respectively. Possible fuel cell reactions under O_2_ and H_2_ atmosphere can be expressed by Equation (13)

(13) $$\mathrm{Cathode}:\;2{\mathrm{BH}}^{+}+1/2O_{2}+2e^{‐}\to 2B+H_{2}O\;\mathrm{Anode}:\;2B+H_{2}\to 2{\mathrm{BH}}^{+}+2e^{‐}\;\mathrm{Total}:\;1/2O_{2}+H_{2}\to H_{2}O$$

The difference between the O_2_ reduction (ORR) potential and H_2_ oxidation (HOR) potential corresponds to the open‐circuit potential (OCP) of H_2_/O_2_ fuel cell. The OCP is quite sensitive to the PIL structure, which can mainly be attributed to the potential of the kinetically very slow ORR.

**FIGURE 17:**
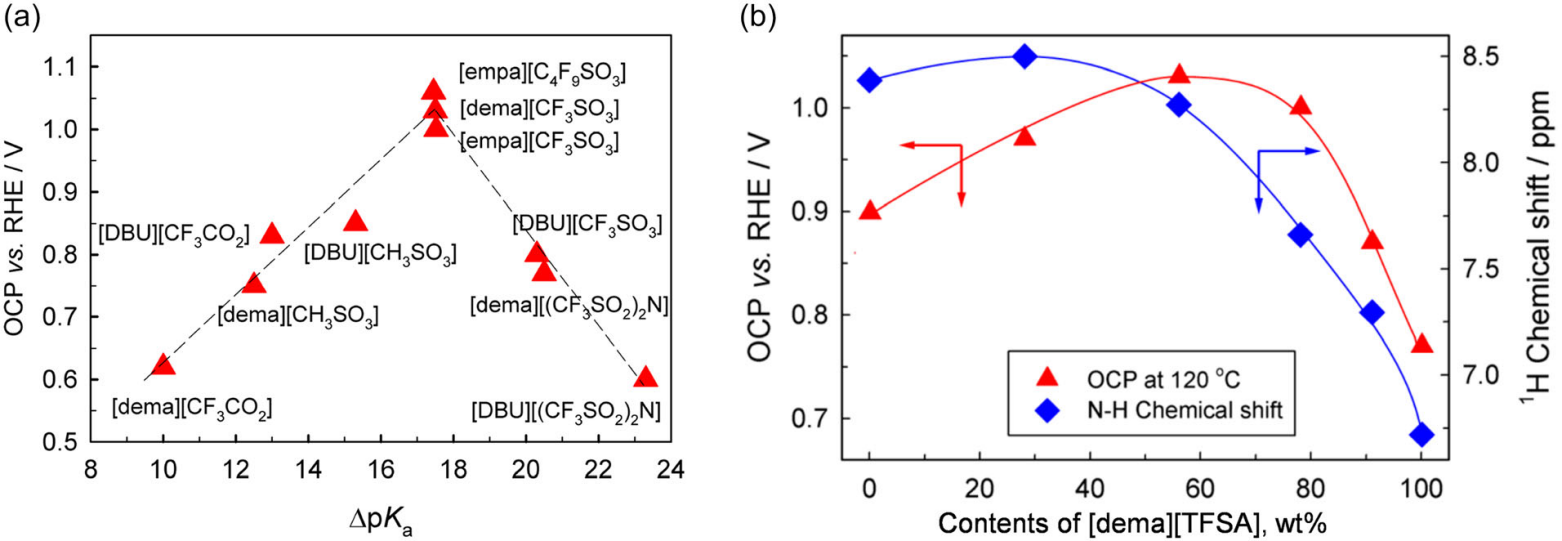
(a) Open‐circuit potential (OCP) for H_2_|O_2_ fuel cells using Pt electrodes in PILs at 150°C as a function of Δ*p* *K*_a_. (b) OCP for H_2_|O_2_ fuel cells using Pt electrodes at 120°C and chemical shift of N‐H proton in [dema] as a function of compositions of [dema][(CF_3_SO_2_)_2_N] and [dema][HSO_4_] binary mixtures. [TFSA]: [(CF_3_SO_2_)_2_N]; [empa]: ethylmethylpropylammonium. Reproduced from (a) Ref. [44] Copyright (2013) with permission from the Royal Society of Chemistry and from (b) Ref. [45] Copyright (2014) with permission from the American Chemical Society.

The OCP is correlated with Δ*p* *K*_a_ of PIL and tends to exhibit a maximum at a certain value of Δp*K*_a_ (Figure 17a) [44]. When Δ*p* *K*_a_ is small, the OCP is lower than 1 V, whereas it becomes higher than 1 V when Δ*p* *K*_a_ is 17–18, especially when the counter anion is [CF_3_SO_3_]. Strong H‐bonds between the cation and anion may hinder facile proton transfer reaction from BH^+^ to O_2_ at low Δ*p* *K*_a_. Another possible reason for the low OCP is electrode poisoning by trace amounts of neutral amine [44]. A further increase in Δ*p* *K*_a_ (>17–18) again lowers the OCP. Under these conditions, the H bonds between the cation and anion become weaker, which results in a stronger N–H bond, as discussed in Section 3. The stronger N–H bond may retard the ORR. Interestingly, when the electrolytes are mixtures of [dema][HSO_4_] (Δ*p* *K*_a_ = 13.5, lower than the optimum Δ*p* *K*_a_) and [dema][(CF_3_SO_2_)_2_N] (Δ*p* *K*_a_ = 20.5, higher than the optimum Δ*p* *K*_a_), both the OCP and the ^1^H chemical shift of NH^+^ proton (*δ*) (a measure of H‐bond strength) exhibit a maximum at certain compositions, as shown in Figure 17b [45]. This result indicates that, in these PIL mixtures, the anions are exchangeable on the time scale of the electrochemical reactions and that the H‐bond strength is averaged by this anion exchange.

**FIGURE 18:**
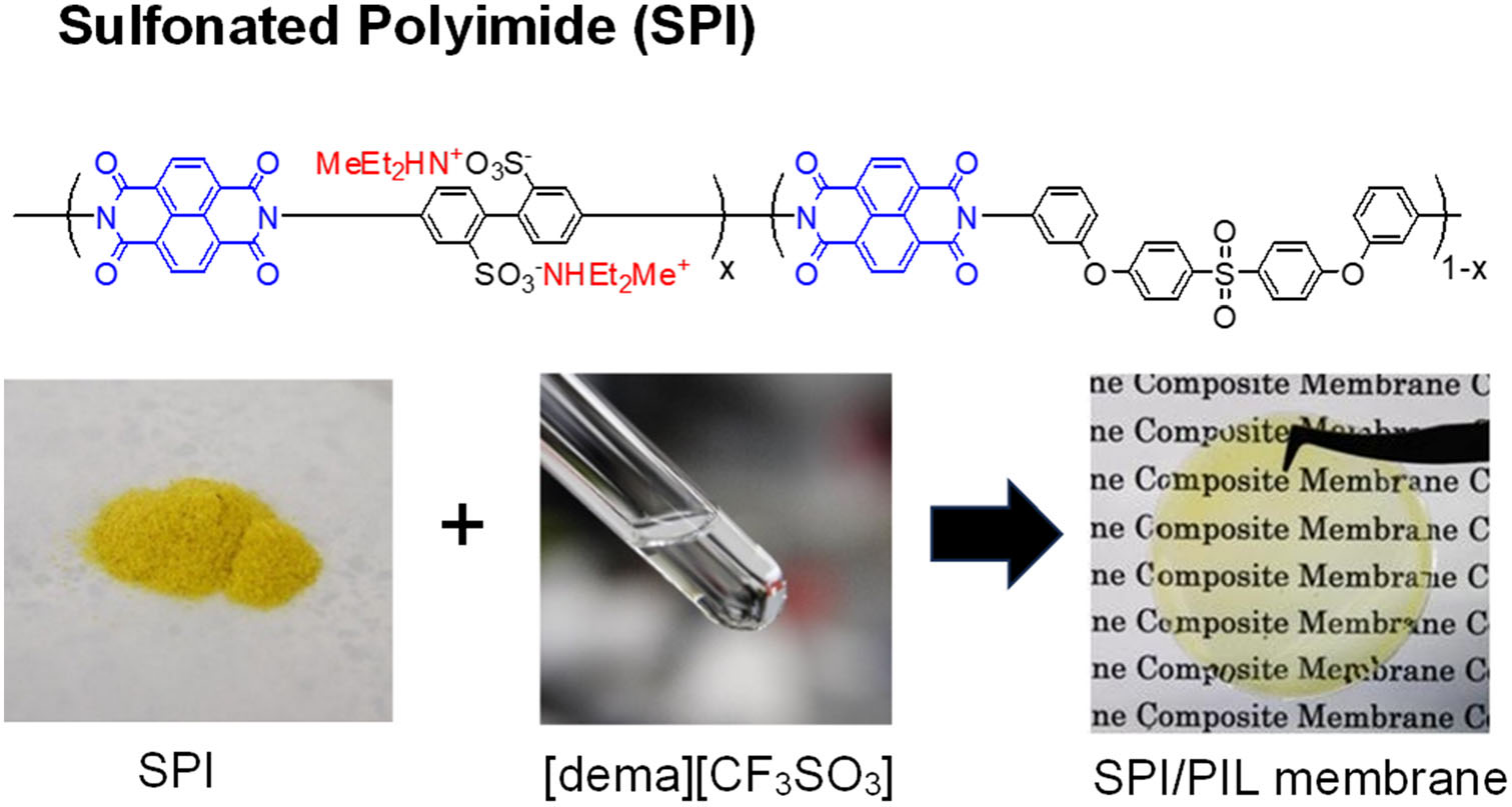
Chemical structure of sulfonated polyimide (SPI) and photos of the formation of SPI/PIL ([dema][CF_3_SO_3_]) membrane.

To design a practical fuel cell using [dema][CF_3_SO_3_], we searched for polymer electrolyte membranes incorporating [dema][CF_3_SO_3_ [46, 47] . However, it was difficult to find a polymer membrane that is compatible with [dema][CF_3_SO_3_] and simultaneously exhibits high mechanical strength and high thermal stability suitable for fuel cell operation under nonhumidified conditions above 100°C. As shown in Figure 18, we found that a sulfonated polyimide (SPI) bearing [dema] as the counter cation of the sulfo groups is compatible with [dema][CF_3_SO_3_] and forms homogeneous composite membranes [47]. Polyimides are thermally stable engineering plastics; however, conventional polyimides are not compatible with [dema][CF_3_SO_3_]. Likewise, the polyimide in Figure 18 without sulfo groups is not compatible with [dema][CF_3_SO_3_]. [dema][CF_3_SO_3_] is preferentially incorporated into the ionic domains of SPI, and the composite membranes exhibit a microphase‐separated structure consisting of ionic and nonionic domains [47]. The maximum amount of [dema][CF_3_SO_3_] that can be incorporated into SPI reaches 75–80 wt%. Even at a high [dema][CF_3_SO_3_] content of 75 wt%, the [dema][CF_3_SO_3_]–SPI composite membranes exhibit high ionic conductivity (*σ* > 10^−^^2^ S cm^−^^1^ at > 100°C), high thermal stability (*T*_d_ > 300°C), and favorable mechanical properties (elastic modulus > 10 MPa, elongation at break = 1.6, tensile strength at break = 10 MPa at room temperature) [47]. These properties arise from the bicontinuous morphology, in which the ionic domains ensure high ionic conductivity and the nonionic domains provide favorable mechanical properties, as revealed by atomic force microscopy, dynamic mechanical analysis, and small‐angle X‐ray scattering measurements [47, 48, 49]. Owing to these favorable properties, IL–SPI composite membranes have been applied not only to fuel cells [49, 50, 51] but also to CO_2_ separation membranes [46, 47, 48] and ionic polymer actuators [49, 51, 52].

**FIGURE 19:**
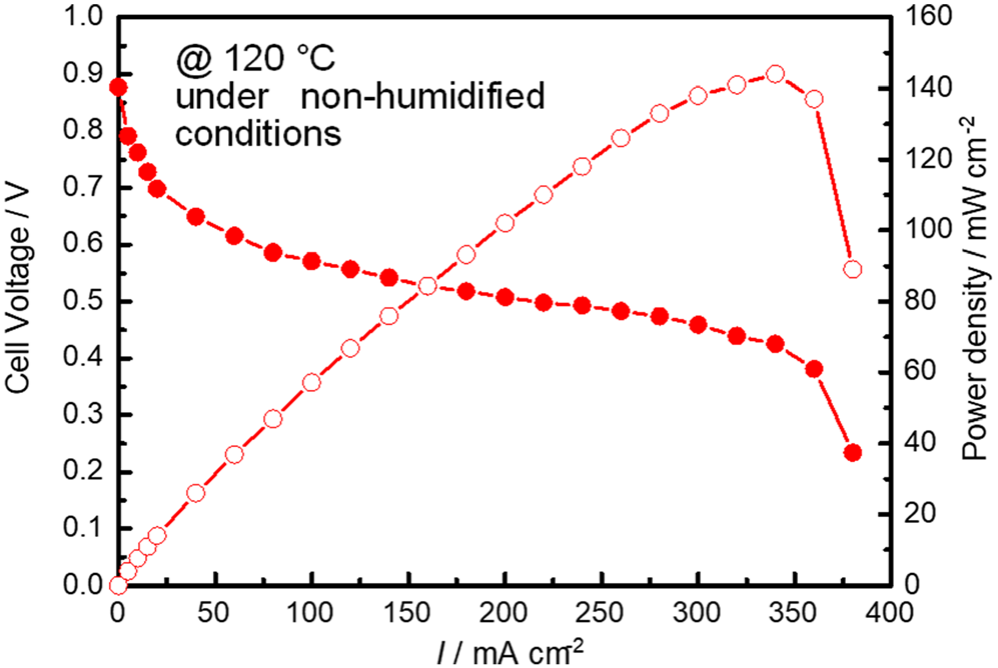
Polarization curve and power density of an H_2_/O_2_ polymer electrolyte membrane (PEM) fuel cell employing an SPI/[dema][CF_3_SO_3_] composite membrane under nonhumidified conditions at 120°C. Reproduced from Ref. [53] Copyright (2021) with permission from The Chemical Society of Japan.

Figure 19 shows the polarization curves of an H_2_/O_2_ polymer electrolyte membrane (PEM) fuel cell employing a [dema][CF_3_SO_3_]–SPI composite membrane under nonhumidified conditions at 120°C [47, 53]. The membrane–electrode assembly (MEA) was fabricated by simply sandwiching the composite membrane between two gas‐diffusion electrodes (30% Pt on Vulcan XC‐72, 0.5 mg cm^−2^, ionomer‐free) [47]. The maximum current density and maximum power density are 400 mA cm^−2^ and 150 mW cm^−2^, respectively. It should be noted that this fuel cell can be operated under nonhumidified conditions at temperatures above 100°C, where conventional PEM fuel cells, which typically employ Nafion membranes, cannot be operated.

**FIGURE 20:**
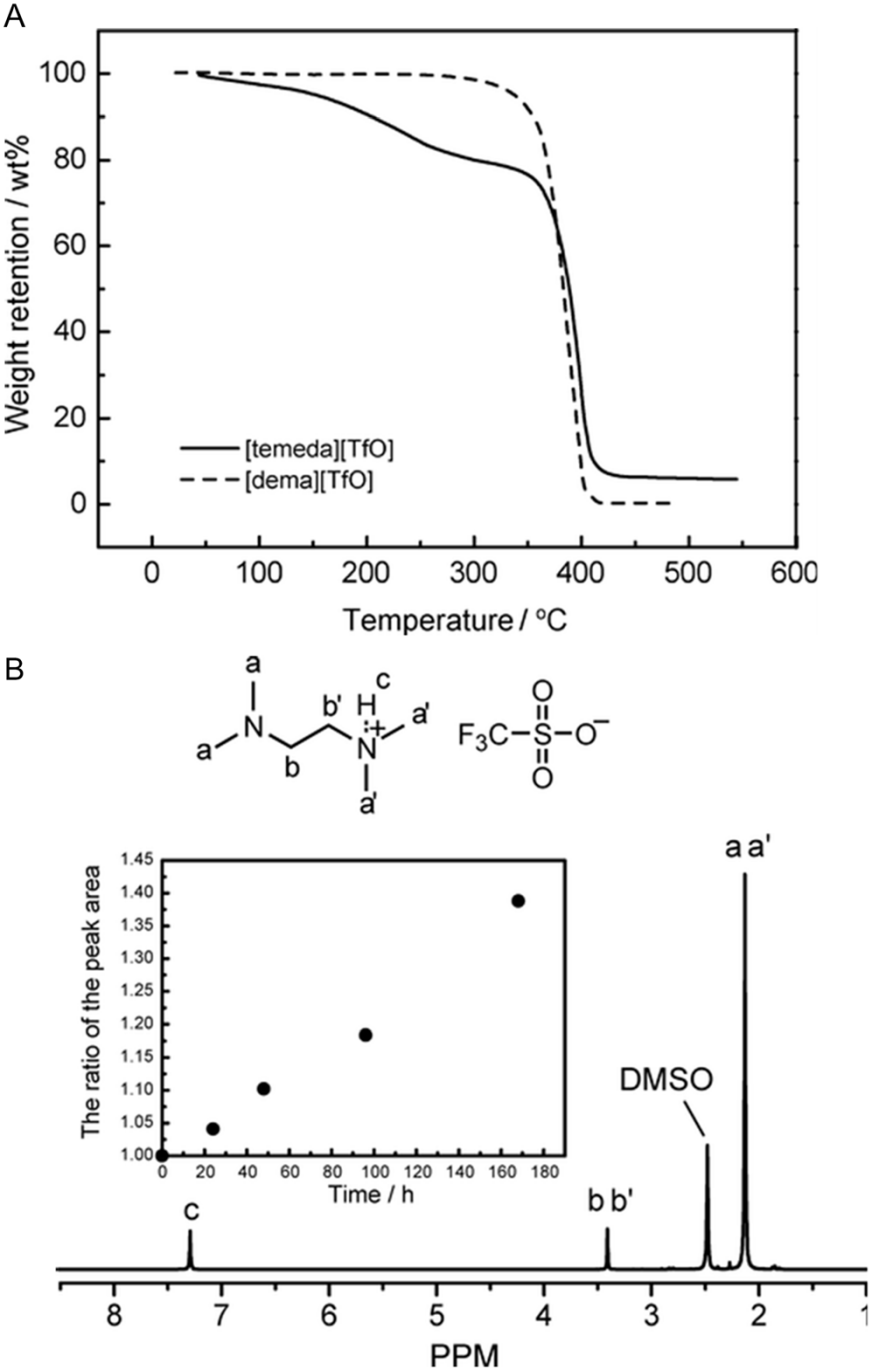
(A) Thermogravimetric curves of [temeda][CF_3_SO_3_] and [dema][CF_3_SO_3_]; (B) ^1^H NMR spectrum of [temeda][CF_3_SO_3_]. The inset shows a change in the ratio of 1/12 (corresponding to the single proton) of the integrated methyl protons (a + a′) to the integrated ammonium proton (c) when [temeda][CF_3_SO_3_] is evacuated at 120°C. Reproduced from Ref. [47] Copyright (2010) with permission from the American Chemical Society.

Proton conduction in the [dema][CF_3_SO_3_]–SPI membranes occurs mainly via migration of the [dema] cation (vehicular mechanism) [47]. Equation (13) indicates that the free base is generated at the cathode as a result of the ORR, and that this free base should then diffuse to the anode, where it accepts protons for the HOR. Judging from the diffusivity, it takes roughly 10^2^ s for the free base to diffuse over a distance of 100 μm. Because the free base is thermally quite unstable, it readily evaporates, resulting in decomposition of the electrolyte. Nevertheless, the fuel cell can be stably operated for at least 200 h [47]. This observation indicates that a proton‐exchange mechanism between the free base and the [dema] cation contributes to proton transport in the operating fuel cell. To confirm this possibility, we prepared a model compound, *N*,*N*‐dimethylaminoethyl‐*N*′,*N*′‐dimethylammonium trifluoromethanesulfonate ([temeda][CF_3_SO_3_]) [47]. [temeda][CF_3_SO_3_] contains both ammonium and amine groups within the same cationic structure, which mimics the situation in the protic IL under fuel cell operation, namely the coexistence of the [dema] cation and its free base. Figure 20A shows the TG curves of [temeda][CF_3_SO_3_] and [dema][CF_3_SO_3_] [47] . The weight loss of [dema][CF_3_SO_3_] starts at ca. 350°C, whereas that of [temeda][CF_3_SO_3_] begins at ca. 100°C, and a second weight loss is observed at ca. 350°C. [temeda][CF_3_SO_3_] is formed by the combination of an amine and a superstrong acid, and its Δ*p* *K*_a_ is similar to that of [dema][CF_3_SO_3_]. The first weight loss corresponds to the amount of *N*,*N*,*N*′,*N*′‐tetramethylethylenediamine generated via an intramolecular proton‐transfer reaction to form the corresponding diammonium cation. The generated diamine easily evaporates because of its relatively low boiling point (*T*_b_ = 122°C). Figure 20B shows the ^1^H NMR spectrum of [temeda][TfO] together with the assignment of its protons [45]. It is interesting to note that the methyl protons assigned to (a) and (a′) cannot be distinguished on the NMR time scale, indicating a fast proton‐exchange reaction between the ammonium and amine groups. The inset of Figure 20 shows the time evolution of the ratio of 1/12 (corresponding to a single proton) of the integrated methyl proton signals (a + a′) to the integrated ammonium protons (c) when [temeda][TfO] is heated at 120°C under vacuum. A ratio of unity (1.0) indicates that one cation carries one ammonium proton. The ratio gradually increases and reaches 1.4 after 180 h, indicating that the fraction of diammonium cations progressively increases during vacuum heating. These results strongly suggest that a proton‐exchange reaction between the ammonium cation and the free amine is possible when they coexist. Such a proton‐exchange mechanism can support proton transport under fuel cell operating conditions and may accelerate proton conduction in the membrane.

## 5 Concluding Remarks

AILs and PILs constitute two major classes of ILs. In this account, we have compared the transport properties of AILs and PILs in terms of their ionicity. The ionicity of AILs is mainly governed by the Lewis basicity of the anions, the directionality of interactions determined by the cation structures, and the alkyl chain length on the cations. Increasing the Lewis acidity/basicity, interaction directionality, or alkyl chain length generally decreases the ionicity. For PILs prepared via proton‐transfer reactions between Brønsted acids and Brønsted bases, H‐bonding interactions between the protonated bases and the conjugate‐base anions play an additional crucial role in determining both transport properties and thermal stability. As the Δ*p* *K*_a_ of the Brønsted acid–base pair decreases, these H‐bonding interactions are greatly enhanced, and the thermal stability is reduced due to back proton transfer from the protonated base to the conjugate‐base anions. Consequently, the ionicity of PILs becomes lower than that of AILs, particularly for PILs with small Δ*p* *K*_a_. The labile protons in PILs can be electroactive toward ORR and HOR, which makes PILs attractive as nonaqueous proton‐conducting materials operable at temperatures above 100°C under nonhumidified conditions. Polymer electrolyte membrane fuel cells have been successfully fabricated by selecting polymers compatible with PILs that exhibit high thermal stability and favorable mechanical properties.

## Author Contributions

M.W. conceived and supervised the research, secured research funding, drafted the manuscript, and revised it. S.T. conducted computational studies and reviewed the manuscript.

## Funding

This study was supported by Japan Society for the Promotion of Science, Japan Science and Technology Corporation, and New Energy and Industrial Technology Development Organization.

## Conflicts of Interest

The authors declare no conflicts of interest.

## Data Availability

The data that support the findings of this study are available from the corresponding author upon reasonable request.
